# Prenatal Parental Separation and Body Weight, Including Development of Overweight and Obesity Later in Childhood

**DOI:** 10.1371/journal.pone.0119138

**Published:** 2015-03-16

**Authors:** Lena Hohwü, Jin Liang Zhu, Lise Graversen, Jiong Li, Thorkild I. A. Sørensen, Carsten Obel

**Affiliations:** 1 Department of Public Health, Aarhus University, Aarhus, Denmark; 2 Institute of Preventive Medicine, Bispebjerg and Frederiksbjerg Hospitals, Copenhagen, Denmark; 3 Novo Nordisk Foundation Center for Basic Metabolic Research, Faculty of Health and Medical Sciences, University of Copenhagen, Copenhagen, Denmark; The University of Kansas Medical Center, UNITED STATES

## Abstract

**Background:**

Early parental separation may be a stress factor causing a long-term alteration in the hypothalamic-pituitary-adrenal-axis activity possibly impacting on the susceptibility to develop overweight and obesity in offspring. We aimed to examine the body mass index (BMI) and the risk of overweight and obesity in children whose parents lived separately before the child was born.

**Methods:**

A follow-up study was conducted using data from the Aarhus Birth Cohort in Denmark and included 2876 children with measurements of height and weight at 9-11-years-of-age, and self-reported information on parental cohabitation status at child birth and at 9-11-years-of-age. Quantile regression was used to estimate the difference in median BMI between children whose parents lived separately (n = 124) or together (n = 2752) before the birth. We used multiple logistic regression to calculate odds ratio (OR) for overweight and obesity, adjusted for gender, parity, breast feeding status, and maternal pre-pregnancy BMI, weight gain during pregnancy, age and educational level at child birth; with and without possible intermediate factors birth weight and maternal smoking during pregnancy. Due to a limited number of obese children, OR for obesity was adjusted for the a priori confounder maternal pre-pregnancy BMI only.

**Results:**

The difference in median BMI was 0.54 kg/m^2^ (95% confidence intervals (CI): 0.10; 0.98) between children whose parents lived separately before birth and children whose parents lived together. The risk of overweight and obesity was statistically significantly increased in children whose parents lived separately before the birth of the child; OR 2.29 (95% CI: 1.18; 4.45) and OR 2.81 (95% CI: 1.05; 7.51), respectively. Additional, adjustment for possible intermediate factors did not substantially change the estimates.

**Conclusion:**

Parental separation before child birth was associated with higher BMI, and increased risk of overweight and obesity in 9-11-year-old children; this may suggest a fetal programming effect or unmeasured difference in psychosocial factors between separated and non-separated parents.

## Introduction

Exposure to stress during fetal life presumably impacts on the susceptibility to develop overweight and obesity [1,2]. The stress hormone cortisol can pass the placental barrier and excessive cortisol may have programming effects on the development of the metabolic function of the fetus [3,4], influencing the appetite regulation later in life [5].

We have earlier found that schoolchildren born to mothers who experienced loss of a close relative during the prenatal time period had an increased risk of overweight [6]. Bereavement, though rare, is considered one of the most stressful life events [7,8]. Previous studies of stressful life events such as maternal distress, parental separation or divorce and neglect, and childhood overweight have shown inconsistent associations if exposed during pregnancy [9,10], no association if exposed within the first six months postpartum [11], and statistically significant associations if exposed during childhood [12–14]. Thus, further studies are needed to clarify the relationship between various prenatal stress exposures and later development of overweight and obesity in children.

The effect of early parental separation may lead to detectable, though mild, long-term alterations in the hypothalamic-pituitary-adrenal axis activity [15]. We hypothesized that exposure to a moderately stressful life event such as parental separation during fetal life will increase the risk of overweight in the offspring. We aimed to examine the association between body mass index (BMI) and the risk of overweight and obesity in children whose parents separated before the child was born.

## Method

### Study population and data collection

We conducted a follow-up study using data from the Aarhus Birth Cohort (ABC). A total of 8719 Danish-speaking pregnant women were included in the ABC at their first antenatal visit between 1 August 1989 and 30 September 1991 at the Department of Obstetrics and Gynaecology, Aarhus University Hospital in Denmark [16]. Around the 30 weeks of gestation, the women were asked to complete two questionnaires on psychological distress and life events experienced during pregnancy, including a question of whether the mother lived together with the father of the unborn child. Information on delivery and the newborn, such as birth weight and gestational age, was obtained from structured birth registration forms filled in by the attending midwife immediately after delivery. The registration forms were manually checked and compared with the medical record by a research midwife before the data entry.

**Fig 1:**
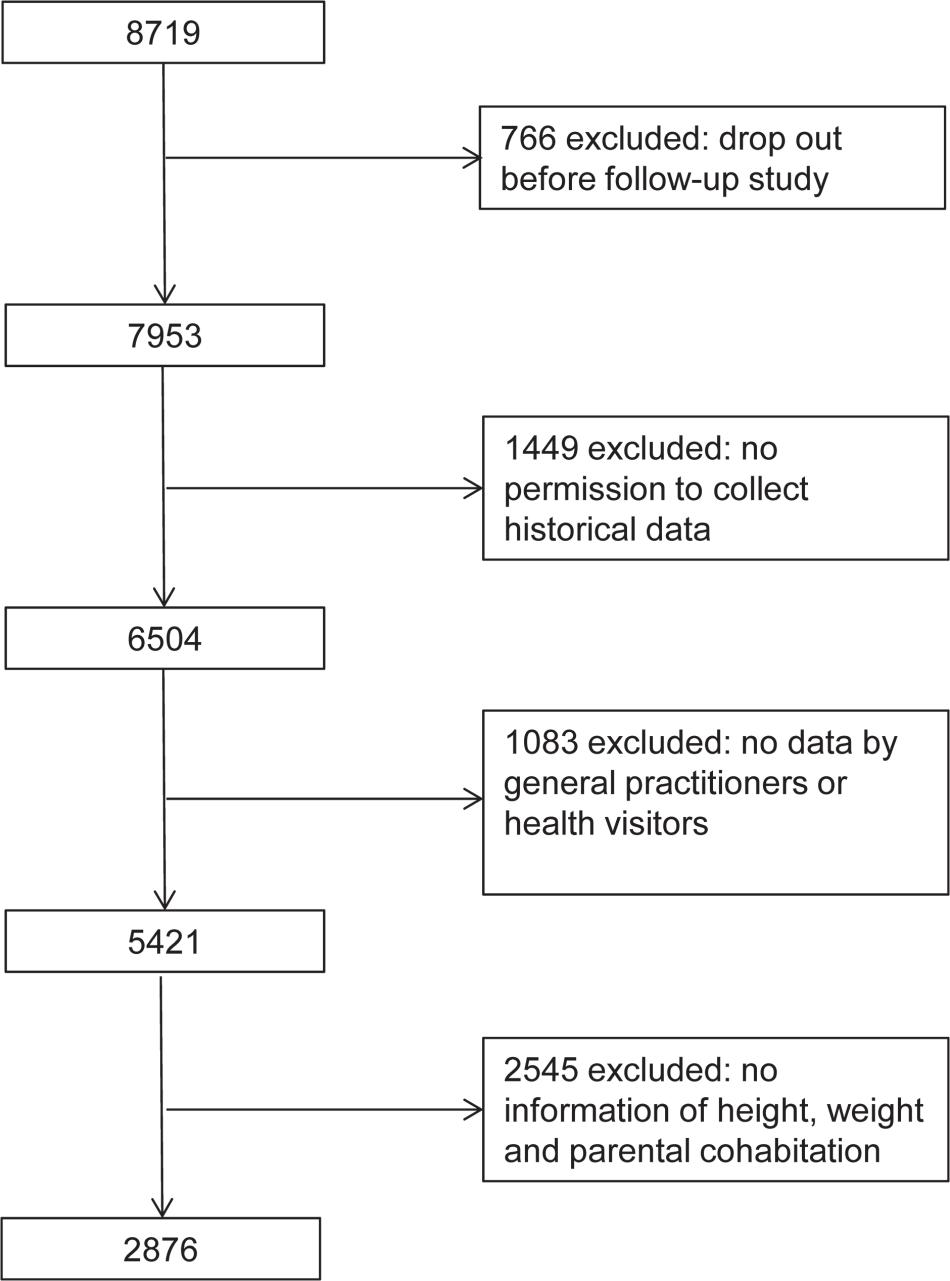
Flow chart of the inclusion of the study population.

In 2001 when the children were 9 to11years old, invitations were sent to participate in a follow up study. A total of 7953 mother-child pairs received a child health questionnaire including a question on whether the biological parents of the child lived together. The parents were also asked to give written consent to collect historical data on the child; permission was granted by 6504 mother-child pairs. In the period from 2010 to March 2012 information on height and weight of 5421 children was collected by general practitioners and health visitors. The number of measurements varied but all children had a least one measurement and two children had 28 measurements collected from birth to the age of 22 years. Finally, we had 2876 singletons for analysis with one measurement under standardized conditions at 9 to 11 years-of-age, and with complete self-reported information on parental cohabitation at the time of birth and at the age of 9 to11 years. A flow chart of selection of the study population is shown in Fig. 1.

### Measures

The exposure variable *parental cohabitation* was dichotomized into children whose biological parents lived together before birth (*Cohabiting*, n = 2752) and children whose biological parents lived separately before birth (*Separate*, n = 124) with *Cohabiting* as the reference group.

The outcome variable was defined by BMI. Further, BMI was categorized as normal weight, overweight including obesity, and obesity, according to the standardized age and gender-specific cut-off points defined by the International Obesity Task Force [17]. BMI z-scores were calculated using the 1990 British Growth Reference as the reference data [18].

Information on potential confounders and covariates was collected from medical records or questionnaire: gender (boy/girl), self-reported maternal pre-pregnancy BMI (kg/m^2^), maternal weight gain during pregnancy (<5, 5–9, 10–14, 15–19, ≥ 20 kg), self-reported maternal educational level (≤ 9, 10–11, ≥ 12 years), maternal age at birth, parity (0, 1, ≥ 2), and breastfeeding status (mean months). Information of the possible intermediate factors, birth weight (< 2500, 2500–3249, 3250–4000, >4000g) and self-reported maternal smoking during pregnancy (0, 1–4, 5–9, 10–14, ≥ 15 cigarettes per day), was also collected.

### Statistical analysis

Data analyses were conducted in Stata version 11.0 (StataCorp LP, College Station, TX, USA).

Descriptive characteristics between *Separate* and *Cohabiting* were tested using chi-square test. The distribution of the BMI data is positively skewed and therefore quantile regression analysis was used to estimate the median BMI with corresponding 95% confidence intervals (CI) in 9 to11 years old children whose parents lived together or separately before the time of birth. The difference in median BMI between *Separate* and *Cohabiting* was tested by Wilcoxon rank-sum test. As BMI z-scores often are used in pediatric studies, we also estimated the mean BMI z-scores and the difference was tested by two-sample t-test with unequal variance in *Separate* and *Cohabiting*. We found no indication of effect modification by gender, and the two genders were thus analyzed together. Further, we found no statistical interaction between parental separation and maternal educational level or smoking during pregnancy; thus, no stratified analyses were made. Significance of interaction was tested using the likelihood-ratio test.

Multiple logistic regression analysis was performed estimating the odds ratio (OR) with corresponding 95% CI for overweight and obesity in children whose parents lived separately before birth. As birth weight and smoking were considered possible intermediate factors rather than confounders, we performed adjusted analyses with and without birth weight and maternal smoking during pregnancy. The OR for overweight was adjusted for gender, maternal pre-pregnancy BMI, maternal weight gain during pregnancy, maternal age and educational level at birth, parity, and breastfeeding status. The OR for obesity was adjusted for the a priori selected confounder maternal BMI only due to a limited number of obese children. A p value of < 0.05 was considered statistically significant.

The follow-up study in 2001 was approved by the Danish Central Ethical Committee (No C-2000-15, ÅA 20000094).

## Results

The descriptive characteristics of the study population are presented in Table 1. The *Separate* group was characterized by younger maternal age and a higher pre-pregnancy BMI, more smokers, lower maternal educational level, and more first-time mothers than the *Cohabiting* group.

**Table 1:** Descriptive characteristics of the study population.

	Separated (n = 124)		Cohabiting (n = 2752)		*P* value^a^
**Mean age; years (SD)**	11.3	(0.7)	11.1	(0.7)	0.05
**Gender; n (%)**					
boys	59	(47.6)	1358	(49.3)	
girls	65	(52.4)	1394	(50.7)	0.70
**Birth weight; n (%)**					
< 2500 gram	5	(4.5)	90	(3.7)	
2500–3249 gram	36	(32.1)	604	(24.9)	
3250–4000 gram	58	(51.8)	1,348	(55.5)	
> 4000 gram	13	(11.6)	385	(15.9)	0.27
**Gestational age; n (%):**					
< 37 weeks	5	(4.4)	110	(4.2)	
≥ 37 weeks	108	(95.6)	2,479	(95.8)	0.93
**Mean maternal pre-pregnancy BMI; kg/m** ^**2**^ **(SD)**	21.9	(4.0)	21.7	(3.2)	0.42
**Maternal weight gain during pregnancy; n (%):**					
< 5 kg	2	(1.9)	27	(1.2)	
5–9 kg	21	(20.2)	235	(10.4)	
10–14 kg	39	(37.5)	932	(41.2)	
15–19 kg	25	(24.0)	787	(34.8)	
≥ 20 kg	17	(16.4)	279	(12.4)	0.01
**Maternal smoking during pregnancy; n (%):**					
0 cigarettes per day	47	(40.1)	1918	(70.9)	
1–4 cigarettes per day	7	(6.0)	121	(4.5)	
5–9 cigarettes per day	14	(12.0)	246	(9.1)	
10–14 cigarettes per day	20	(17.1)	264	(9.7)	
≥ 15 cigarettes per day	29	(24.8)	156	(5.8)	< 0.01
**Maternal educational level at child birth; n (%):**					
≤ 9 years	43	(48.9)	617	(25.9)	
10–11 years	14	(15.9)	751	(31.5)	
≥12 years	31	(35.2)	1015	(42.6)	<0.01
**Mean maternal age at child birth; years (SD)**	29.1	(5.8)	30.0	(4.5)	0.04
**Mean breast feeding; months (SD)**	4.2	(2.5)	4.2	(2.1)	0.94
**Parity; n (%):**					
0	72	(58.1)	1348	(49.0)	
1	35	(28.2)	998	(36.3)	
≥ 2	17.0	(13.7)	403	(14.7)	0.12
**Overweight children; n (%)**	27	(21.8)	357	(13.0)	0.01
**Obese children; n (%)**	6	(4.8)	44	(1.6)	0.01

Separate: Children whose biological parents lived separately before birth.

Cohabiting: Children whose biological parents lived together before birth.

SD standard deviation.

^a^ Chi-square test.

BMI body mass index.

The median and mean BMI z-scores in the *Cohabiting* and *Separate* groups, respectively are shown in Table 2. The median BMI was statistically significantly higher in children whose parents lived separately (17.84 kg/m^2^, 95% CI: 17.35; 18.43) than in children whose parents lived together (17.36, 95% CI: 17.28; 17.47) before birth of the child, p = 0.02. The mean BMI z-scores were insignificantly higher in the *Separate* group (0.26, 95% CI: 0.04; 0.48) than the *Cohabiting* group (0.10, 95% CI: 0.06; 0.14), p = 0.18.

**Table 2:** Median and mean body mass index z-scores (kg/m^2^) in 9 to 11 years old children whose parents lived separately and together before birth of the child.

	Cohabiting (n = 2752)		Separate (n = 124)		*P* value
Median (95% CI)	17.36	(17.28; 17.47)	17.84	(17.35; 18.43)	0.02^a^
Z-scores (95% CI)	0.10	(0.06; 0.14)	0.26	(0.04; 0.48)	0.12^b^

CI denotes confidence interval.

^a^ Wilcoxon rank-sum (Mann-Whitney) test.

^b^ Two sample t-test with unequal variance.

Cohabiting: Children whose biological parents lived together before birth.

Separate: Children whose biological parents lived separately before birth.

**Fig 2:**
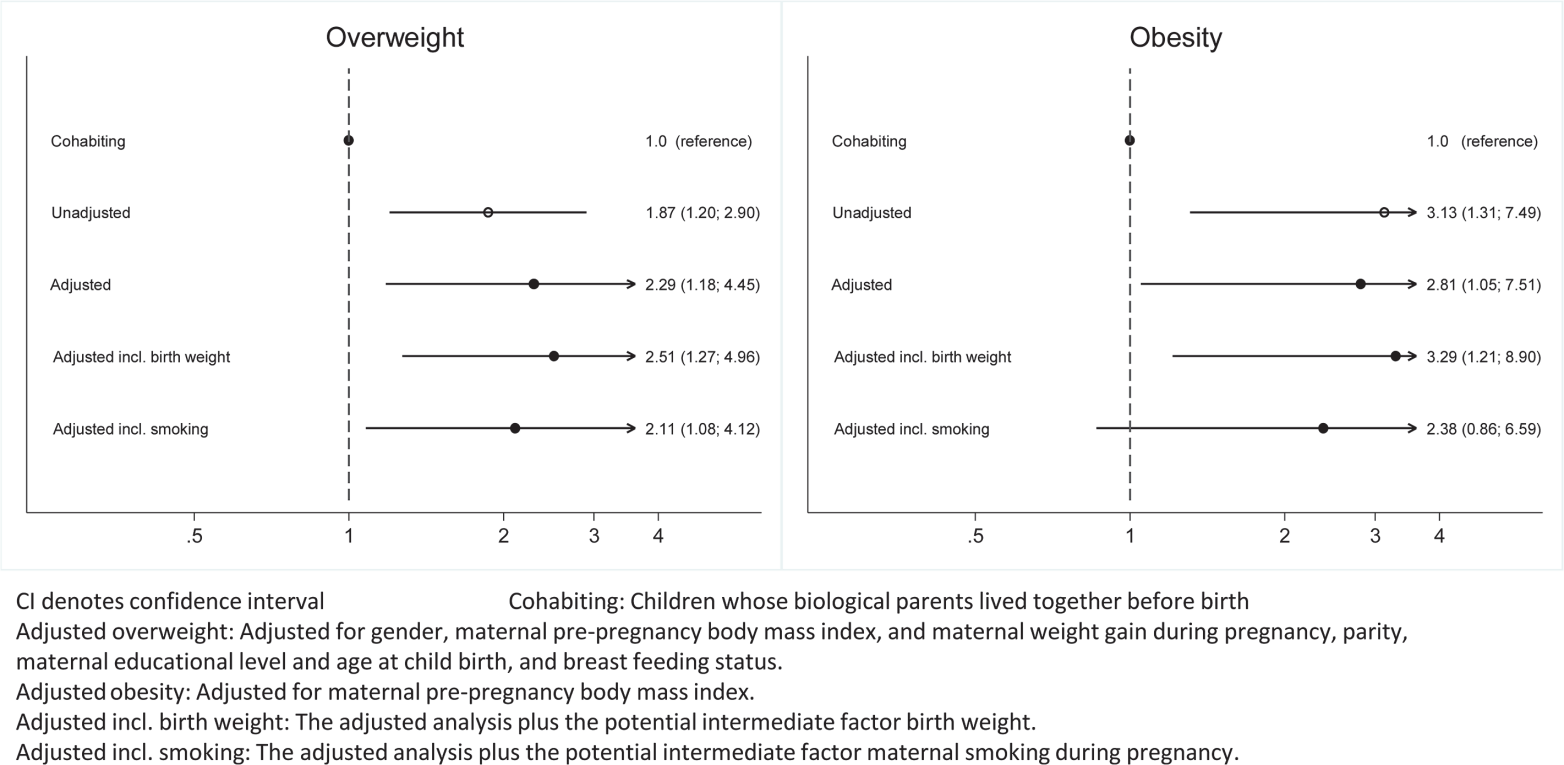
Raw and adjusted odds ratio (95% confidence intervals) for overweight and obesity in children whose parents were separated before birth (n = 2876).

Fig. 2 shows the raw and adjusted ORs for overweight and obesity with and without birth weight and maternal smoking during pregnancy. The risk of overweight was two-fold higher in children whose parents lived separately before birth (adjusted OR 2.29, 95% CI: 1.18; 4.45). The OR for obesity was 2.81 (95% CI: 1.05; 7.51) for children of parents who lived separately before birth. Adjustment for the possible intermediate factors, birth weight and maternal smoking during pregnancy, did not produce substantial changes to the estimates for overweight and obesity, although OR for obesity became non-significant when adjusting for maternal smoking during pregnancy (Fig. 2).

## Discussion

We found significantly higher median BMI in 9 to11years old children whose parents lived separately before the time of birth compared with children whose parents lived together before time of birth. We found that parental separation before birth was significantly associated with increased risk of overweight and obesity in the offspring.

### Comparison with other studies

Our finding of a higher median BMI in children whose parents lived separately before childbirth could reflect a change in the right tail of the BMI distribution. Therefore, we also focused on the risk of overweight and obesity where our findings are in line with other studies [12,13]. Two longitudinal studies involving American children also addressed the effects of parental separation, but later in the life of the child. One study found an 83% increased odds of overweight in 3 to 5 years old children living in families where the parents were separated [12]. In another study, the risk of obesity in 5 to 14 years old children in a period of two years before parents’ separation was examined [13], and this study showed that children who became obese in the years before the separation were more likely to remain obese in the future [13]. As we examined the exposure before the birth these findings could not be compared to our study.

We have previously found that schoolchildren born to mothers who experienced bereavement during the prenatal time period had an increased risk of childhood overweight at the age of 12 years (adjusted OR 1.68, 95% CI: 1.08; 2.61) [6]. In the present study, we found a higher risk for overweight in 9 to 11 years old children of parents that lived separately before the child was born, adjusted OR 2.29 (95% CI: 1.18; 4.45). This could indicate that separation may be an exposure with a long-term impact (during childhood), and that the stress exposure elicited by bereavement may be somewhat unclear.

Two previous Danish studies have examined self-reported maternal distress (anxiety, depression, or stress) either during pregnancy or postpartum and risk of overweight in 7 years old children [9,11]. One study found a 9% increase of overweight, though not statistically significant, in the offspring to mothers reporting distress around gestational week 30 [9]; the other study showed no association between risk of overweight in offspring to mothers reporting distress six months postpartum [11]. In our study, the risk of overweight was higher in children of parents who lived separately before birth of the child. These children were, however, older than those in the two above-mentioned Danish studies. If there is a long-term programming effect during pregnancy, we would expect the risk to be higher in the older children.

### Strengths and limitations

We believe the use of height and weight measured under standardized conditions, and the rating of parental separation as a moderately stressful life event [7], are strengths in our study. However, our study has some limitations. First, we cannot rule out selection bias, as 2876 of the 8719 participants in the ABC comprised the study population in the present study. If there were a higher proportion of separated parents with overweight and obese children among the non-participants, we may have underestimated the risks. Secondly, the timing of self-reported separation is not measured and stress might have happened before the conception as separation is usually a process that begins long before ending with a legal divorce [19]. Therefore, mediating factors of both pre-separation stress (e.g. conflict with the spouse) and post-separation stress (e.g. continuing conflict with the ex-spouse, economic decline) may be present. Most likely it remains unknown how much timing has biased the results. Third, we had no information on life style, e.g. dietary nutrient intake. The total calorie and saturated fatty acid intakes have shown to be higher in single-parent than dual-parent households [20], and we may therefore have overestimated the association. Further, we also had no information on the pubertal status of the children. The pubertal growth spurt begins earlier in girls than boys, and peak velocity for weight occurs about 6 months before peak velocity for height [21]. If more children in the *Separate* group begin puberty growth spurt earlier, we may have overestimated the association. Moreover, maternal smoking during pregnancy may be considered a confounder rather than an intermediate factor. However, the association between parental separation before birth and the risk of overweight and obesity slightly changed, and it did not alter the conclusion. Finally, biological parents to an unborn child may have chosen to live separately for practical reason. This could be the case in situations where one of the parents has a long-distance job that geographically requires them to live apart or a single mother having a child with an anonymous donor. We had no available data on such cases, but if they caused less stress we may have underestimated the association between prenatal stress exposure due to parental separation and risk of overweight and obesity in offspring.

### Biological or societal mechanisms

Examining the exposure during pregnancy may predominantly be exploring a potential programming effect rather than interfering in children’s active coping skills that may change through childhood. A biological mechanism prenatally may be that the placenta enzyme 11β-hydroxysteroid dehydrogenase type 2 during normal conditions acts as a fetal-placental barrier preventing the majority (80–90%) of maternal glucocorticoid cortisol from crossing the placenta in an active form [22,23]. As the concentration of cortisol in the fetus is correlated to maternal level of cortisol during pregnancy [24], increased cortisol caused by prenatal stress can lead to down-regulation of the 11β-hydroxysteroid dehydrogenase type 2, which has been shown in both human [25] and animal [26,27] studies. Consequently, an increased concentration of cortisol in the fetus may influence fetal development of the hypothalamic-pituitary-adrenal axis [3,4]. Increased body weight in the offspring has been shown in antenatal maternal nutrient-restricted sheep [28] and stress-responded maternal stress-sensitive mice [29]. This could be explained by transcript and epigenetic changes in the hypothalamus, the part of the brain regulating appetite and food intake, related to increased susceptibility to overweight and obesity [5]. As a result, altered programming effect of growth, although mild, in the offspring may be established [15,30].

Societal mechanism may be an alternative explanation. The impact of separation is potentially complicated by a multitude of potential environmental factors during the process [31]. In adults, separation may not only have a negative outcome (such as a personal tragedy) but also positive outcomes such as relief and an opportunity for personal growth [19]. Even though the stressful environment in the time leading up to the separation is replaced by a more harmonious atmosphere after the separation, there may be variations of interactions and events that precede and follow possibly influencing the child’s mental and physical health such as decline in parental support and less healthy eating patterns [19,32]. The economic consequences of divorce are greater for women than men [19] and socioeconomic differences have been shown to be associated with differences in BMI in childhood, emerging at the age of 4 years and widening with increasing age [33]. Parental separation also affects the parenting style and is associated with more difficulties in raising the children [19,34]. The shared responsibility of the child may influence the child’s social well-being and affect the risk of overweight and obesity. Further, children living permanently together with both their parents may also experience stressful environments, which further challenge a comparison.

In conclusion, parental separation before the birth of the child was associated with higher BMI, as well as increased risk of overweight and obesity in 9 to11 years old children. These findings may indicate a fetal programming effect due to prenatal moderate stress exposure. However, future studies are to estimate whether these programming alterations will persist into adulthood. Alternative, the association may reflect the difference in psychosocial characteristics of separated parents.
